# Sexually transmitted mutualist nematodes shape host growth across dung beetle species

**DOI:** 10.1002/ece3.11089

**Published:** 2024-03-10

**Authors:** Levi W. Burdine, Armin P. Moczek, Patrick T. Rohner

**Affiliations:** ^1^ Department of Biology Indiana University Bloomington Bloomington Indiana USA; ^2^ Department of Ecology, Behavior, and Evolution University of California San Diego La Jolla California USA

**Keywords:** *Diplogastrellus*, dung beetle, mutualism, Scarabaeidae, symbiosis

## Abstract

Many symbionts are sexually transmitted and impact their host's development, ecology, and evolution. While the significance of symbionts that cause sexually transmitted diseases (STDs) is relatively well understood, the prevalence and potential significance of the sexual transmission of mutualists remain elusive. Here, we study the effects of sexually transmitted mutualist nematodes on their dung beetle hosts. Symbiotic *Diplogastrellus monhysteroides* nematodes are present on the genitalia of male and female *Onthophagus* beetles and are horizontally transmitted during mating and vertically passed on to offspring during oviposition. A previous study indicates that the presence of nematodes benefits larval development and life history in a single host species, *Onthophagus taurus*. However, *Diplogastrellus* nematodes can be found in association with a variety of beetle species. Here, we replicate these previous experiments, assess whether the beneficial effects extend to other host species, and test whether nematode‐mediated effects differ between male and female host beetles. Rearing three relatively distantly related dung beetle species with and without nematodes, we find that the presence of nematodes benefits body size, but not development time or survival across all three species. Likewise, we found no difference in the benefit of nematodes to male compared to female beetles. These findings highlight the role of sexually transmitted mutualists in the evolution and ecology of dung beetles.

## Graphical abstract

In this study, we investigate how sexually transmitted symbionts affect life history traits of three species of dung beetle. We found a consistent positive effect of the presence of *Diplogastrellus* nematodes on the size of three relatively distantly related beetle host species. This finding suggests that sexually transmitted mutualist nematodes may play a much larger role in dung beetle life history than currently assumed.

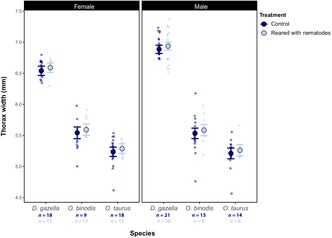

## 1 INTRODUCTION

Symbionts and hosts possess strong potential to influence each other's development and evolution (McFall‐Ngai et al., 2013; Douglas, 2014; Gilbert et al., 2015; Grieneisen et al., 2020). Not surprisingly, both hosts and symbionts have evolved diverse strategies to ensure as well as constrain symbiont transmission across generations (Bright & Bulgheresi, 2010; Correa & Ballard, 2016; Rosenberg & Zilber‐Rosenberg, 2021). One nexus in this dynamic emerges in the physical contact that transpires during copulation and birth. In animals with internal fertilization, the sexual transmission of symbiotic bacteria, fungi, and small animals (e.g., lice: Patel et al., 2021) is common and often causes deleterious effects on host performance, leading to sexually transmitted disease (STD) (e.g., Oriel & Hayward, 1974; Sheldon, 1993). STDs are thought to increase the cost of multiple mating and can thus favor the evolution of reduced mating frequency, profoundly shaping the evolution of mating systems and condition‐dependent sexual signaling (Ashby & Boots, 2015; Hamilton & Zuk, 1982; Kokko et al., 2002; Loehle, 1997). However, although many sexually transmitted infections have deleterious effects, some sexually transmitted symbionts can be beneficial for their hosts (Bhattarai & Stapleton, 2012; Smith & Mueller, 2015). For instance, *Anopheles* mosquitos sexually transfer symbiotic *Asaia* bacteria (Damiani et al., 2008), which, if present, have beneficial effects on mosquito development (Chouaia et al., 2012). Similarly, the bacteria *Hamiltonella defensa* and *Regiella insecticola*, which increase the fitness of their aphid hosts, are sexually transmitted and indirectly passed on from father to offspring (Moran & Dunbar, 2006). Yet, how widespread these sexually transmitted mutualists are, and how they may shape host life history and fitness remains poorly understood (Smith & Mueller, 2015). Here, we study dung beetles and their sexually transmitted nematodes to begin investigating these dynamics.

Due to their nutritional ecology, life history, and ease of manipulation, dung beetles and their symbionts emerge as a useful study system to explore the role of symbiosis in development and evolution (Rohner et al., 2024; Schwab et al., 2016). Adult beetles of many species colonize fresh dung pads by flight and walking, often from considerable distances, and mating takes places inside and underneath dung pads. Adult females then construct underground chambers filled with processed and compacted cow dung (Hanski & Cambefort, 1991). In each of these “brood balls,” females deposit a single egg. Upon hatching, the larva starts to feed on, and manipulate, its brood ball and completes its entire juvenile development inside the brood ball until it emerges as an adult. During oviposition, the female vertically transmits its gut microbiome, including various bacterial and fungal taxa, to its offspring via a fecal pellet (Estes et al., 2013; Shukla et al., 2016). This microbial inoculate is then consumed by the larva and subsequently spread through its entire brood ball. Preventing offspring from receiving these symbionts results in prolonged development, reduced adult size, and reduced fitness (Schwab et al., 2016; Parker et al., 2019; Parker et al., 2021; Rohner et al., 2024). Mutualistic microbes thus play a major role in beetle development and evolution.

In addition to symbiotic gut bacteria and fungi, dung beetles also interact with symbiotic nematodes, as is common in insects in general (e.g., gall‐forming flies: Giblin‐Davis et al., 2001; black scavenger flies: Pont & Meier, 2002; bark beetles: Susoy & Herrmann, 2014). Some of these nematodes are merely phoretic and found underneath the beetle's wing covers (elytra) (Ragsdale et al., 2022). However, Ledón‐Rettig et al. (2018) demonstrated the presence of *Diplogastrellus monhysteroides* (Diplogastridae) nematodes on the genitalia of male and female *Onthophagus taurus* (Scarabaeidae). These nematodes are horizontally transmitted via genital contact and vertically inherited from mother to offspring during oviposition. Once inside the brood ball, nematodes start to dwell in the dung mass and establish large population sizes. As soon as the beetle larva undergoes pupation, nematodes (re)colonize the newly formed adult and are transported out of the brood ball. Once the host reaches sexual maturation, nematode symbionts are then spread in the population via genital contact. Crucially, this symbiosis is not merely commensal. The presence of the nematodes benefits larval growth rate and adult body size, most likely due to their effects on the bacterial and fungal communities in the brood ball (Ledón‐Rettig et al., 2018). Both traits tested are fitness‐related as larger females, for instance, produce more offspring (Hunt & Simmons, 2004). This association between dung beetles and their nematodes thus offers a promising opportunity to explore the contributions of sexually transmitted mutualists in evolution.

Nematodes of the genus *Diplogastrellus* are commonly found on decaying organic matter, including vertebrate dung, decaying vegetation (Sudhaus & Fürst von Lieven, 2003), as well as the insects feeding on these substrates (e.g., sugarcane weevils: Kanzaki et al., 2008; fungus‐growing termites: Kanzaki et al., 2019). *Diplogastrellus monhysteroides* is particularly abundant in horse and cow dung and has been reported from all continents except Antarctica (Kiontke & Sudhaus, 1996; Sachs, 1950). This species is not only found in association with *Onthophagus* dung beetles and its close relatives but also with coprophagous beetles in other scarab subfamilies (such as *Aphodius* spp.) and members of entirely separate beetle families, such as dor beetles (Geotrupidae: *Geotrupes* spp.), rove beetles (Staphylinidae: *Atheta* spp., *Oxytelus* spp.), and hydrophilids (Hydrophilidae: *Cercyon* spp.) (Kiontke & Sudhaus, 1996; Kühne, 1996). The positive effects of its presence on beetle development may thus be more widespread than currently recognized. However, thus far, the beneficial effects of *D. monhysteroides* on insect life history have only been documented in a single study and on a single species of *Onthophagus* dung beetle (Ledón‐Rettig et al., 2018). Here, we therefore sought to (i) assess the repeatability of these mutualist effects in the original study species *O. taurus* and (ii) test whether similar or different effects may be present in ecologically similar but relatively distantly related species of dung beetles. Specifically, we studied the effects of *Diplogastrellus* nematodes on juvenile survival, development time, and body size of *Onthophagus binodis* and *Digitonthophagus gazella* beetles, which diverged from the previously studied *O. taurus* ca. 35 and 40 million years ago, respectively (Breeschoten et al., 2016; Parzer et al., 2018). In addition, (iii) we tested whether the mutualistic effects of *Diplogastrellus* differed between host sexes. Sexes (or sex roles) are often associated with varying strengths of plastic responses to nutritional conditions (Rohner et al., 2018; Stillwell et al., 2010). As the presence of nematodes modifies the microbial environment in the brood ball, we hypothesized that male and female beetles may—given their differential responsiveness to nutritional conditions—also differ in their response to the presence (or absence) of nematodes. While we find evidence of beneficial nematode‐mediated effects on body size, we find little evidence for sex or species differences in the degree of these benefits. Although the observed effects were overall modest, our results suggest that beneficial sexually transmitted symbioses may be more common than currently appreciated, and that uncovering their evolutionary consequences will require further research.

## 2 MATERIALS AND METHODS

### 2.1 Dung beetle husbandry

*Onthophagus taurus* (originally collected in North Carolina, United States), *Onthophagus binodis* (Western Australia, Australia), and *Digitonthophagus gazella* (Florida, United States; and Queensland, Australia) were housed in large plastic containers filled with a mixture of two parts sand and one part soil. Colonies were fed twice a week with ad libitum cow dung. The *O. taurus* and *O. binodis* colonies were held at 24°C while the *D. gazella* colonies were held at 29°C, following protocols established in prior studies (Moczek, 2006; Moczek et al., 2006). All colonies were maintained with 16 h of light and 8 h of darkness.

### 2.2 Identification and maintenance of nematode cultures

*Diplogastrellus* nematodes were collected from the genitalia of multiple *Onthophagus taurus* males, originally collected in North Carolina. Dauer larvae were collected from the male aedeagus and suspended in 1 mL of Dulbecco's phosphate‐buffered saline (DPBS). 10 μL of this solution were then added to 10 Falcon tubes that were each filled with ca. 20 mL of previously frozen cow dung. Each Falcon tube was closed with a lid that contained a mesh for ventilation and incubated at room temperature. To propagate these laboratory cultures, nematodes were rinsed off the cow dung surface with ca. 1 mL of DPBS and transferred onto new Falcon tubes every 10–14 days.

To identify the species of nematode, we sequenced the 18S ribosomal RNA gene following the general protocol of Ledón‐Rettig et al. (2018). In brief, we isolated single nematodes and placed them individually into 200 μL PCR tubes filled with 19 μL of 1XPCR buffer and 1 μL proteinase K (10 mg/mL). Tubes were spun down using a centrifuge and placed at −80°C for 30 min. Genomic DNA was released by heating the sample up to 65°C for 60 min. Proteinase K was deactivated by heating the sample to 95°C for 15 min. 18S rRNA gene was then amplified using the SSU_18A (5′‐AAAGATTAAGCCATGCAT‐3′) and SSU_26R (5′‐CATTCTTGGCAAATGCTTTCG‐3′) primers. All nematodes collected on *O. taurus* aedeagi matched the sequence of *Diplogastrellus monhysteroides* which was previously shown to be in a symbiotic relationship with this and other dung beetle species (Kühne, 1996; Ledón‐Rettig et al., 2018; Sudhaus & Fürst von Lieven, 2003). 18S sequencing was repeated over time to ensure that the laboratory cultures used in experimental assays were not contaminated with other nematode species.

### 2.3 Experimental rearing of dung beetle larvae with and without symbiotic nematodes

To generate experimental animals for each of the three dung beetle species, 6 females and 3 males were randomly selected from each laboratory colony and placed into ovipositing containers. These rectangular containers (27 cm × 17 cm × 28 cm) were filled with a standard sterile mixture of sand and topsoil and topped off with an excess of defrosted cow dung. After 5 days, all containers were deconstructed, and the brood balls were collected. Eggs were removed from their natal brood balls using sterilized forceps. The eggs were then surface sterilized with a 100 μL rinse of a 1% bleach and 0.1% Triton‐X 100 solution followed by two rinses with 1 mL distilled water (Schwab et al., 2016). Once sterilized, the eggs were transferred into artificial brood balls constructed in 12‐well tissue culture plates. Each beetle egg was placed in the center of each well on top of a bed of squeezed cow dung previously described in more detail in Shafiei et al. (2001). The dung used in the construction of these plates was collected from hay‐fed cows, which is more challenging to digest for beetle larvae than the dung of cows fed on grass (Rohner & Moczek, 2021).

Once beetle larvae were added to their artificial brood balls, half of the individuals were inoculated with nematodes. A live nematode solution was created by rinsing nematodes off the surface of three of the previously described *Diplogastrellus* cultures. Nematode cultures were rinsed with about 3 mL of DPBS, and the resulting solution was collected in multiple separate 1.5 mL Eppendorf tubes and spun down in a centrifuge at 224 *g* for 2 min. The pellets (containing nematodes), as well as a 1 mL aliquot of the supernatant (which does not contain nematodes based on visual inspection under a microscope) were saved, and the rest was discarded. The pellets were carefully resuspended in about 200 μL of DPBS and combined into one tube. To calculate the number of nematodes in the sample, we transferred three 20 μL aliquots of this solution separately onto a glass slide and counted the number of live nematodes under a dissecting microscope. We then calculated the average number of nematodes per 20 μL stock solution across the three aliquots and diluted the original stock solution to a concentration of 1 live nematode per 1 μL. Half of the eggs were inoculated with 20 μL of this nematode solution. As a control treatment, we inoculated the other half of all larvae with 20 μL of the (nematode‐free) supernatant set aside previously.

Beetle larvae (with or without nematodes) were incubated at 27°C throughout their juvenile development. This experiment was done in two blocks of data collection. During the first block, individuals were monitored every day and the date each individual hatched and molted into an adult was recorded. During the second block, individuals were monitored once every 2 days. Throughout both blocks, any death prior to adulthood was recorded, and once adulthood was reached (as indicated by the eclosure from the pupal cuticle), individuals were euthanized and stored in 70% ethanol. To measure adult body size, we took calibrated pictures of each individual's thorax in a randomized order using a Pixelink PL‐D797CU‐T camera attached to a Leica MZ‐16 stereomicroscope. Pronotum width (as a suitable estimate of overall body size, see Rohner, 2021) was quantified using ImageJ (Schneider et al., 2012).

### 2.4 Statistical analysis

The effect of nematode presence on beetle body size and eclosure time was analyzed using linear models and analysis of variance (ANOVA). We fitted pronotum width as a function of species, sex, nematode treatment, and all interactions. Non‐significant interactions were removed. To account for potential differences between experimental blocks, experimental block was added as a fixed factor in the analyses. We used similar models to analyze eclosure time, but because this variable was only recorded in the first experimental block, we did not add block in these models. For general linear models, partial eta squared ($\eta_{p}^{2})$ was calculated as an estimate of effect size using the R package *heplots* (Fox et al., 2021). We also ran additional AICc‐based model selection analysis using the function dredge implemented in the R package *MuMIn* (Barton, 2022; see Table S1).

The effect of nematodes on host survival was tested using a generalized linear model with a binomial error distribution. Because larval sex can only be assessed in late larval development (Moczek & Nijhout, 2002), we could not assess sex differences in larval mortality. We used chi‐squared tests to assess the significance of individual predictors. All analyses were conducted using R version 4.2.1 (RCoreTeam, 2020).

## 3 RESULTS

**FIGURE 1:**
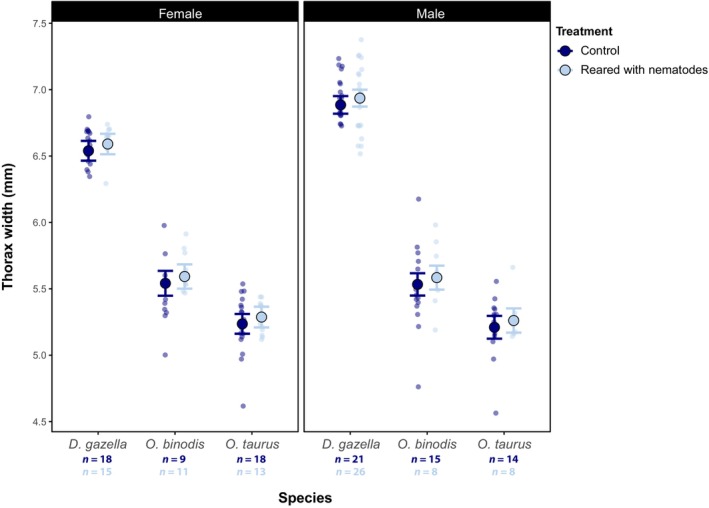
Estimated marginal means of thorax width (body size) across species and treatments for females and males. The effect of the presence of *Diplogastrellus* nematodes in the larval environment on adult size of females (left) and males (right) of three dung beetle species. Error bars indicate standard errors associated with parameter estimates derived from the model with the lowest AICc. Sample sizes (*n*) are given by treatment combination. See Figure S1 for raw data.

In this experiment, we aimed to explore how the presence of sexually transmitted *Diplogastrellus* nematodes affects fitness‐related life history traits in three dung beetle taxa. To do so we assessed the effects of nematodes on host body size, time to eclosure, and juvenile survival. We found a modest yet significant increase in the width of the adult thorax in individuals reared in the presence of nematodes across all three species tested (*F*_1,168_ = 5.57, *p* = .019, $\eta_{p}^{2}$ = 0.02; Table 1a, Figure 1; note that pairwise *t*‐tests between the treatments within sex and species combinations were not significant, however, given the small sample size within these subgroups, this is most likely due to limited statistical power to detect modest effects). We did not detect a significant difference in the degree to which each species was affected by the presence of nematodes (non‐significant treatment‐by‐species interaction: *F*_2,166_ = 1.68, *p* = .189). As these beetle species are relatively distantly related, this suggests that similar positive effects might potentially be found in a large number of species. Furthermore, these positive effects on body size did not differ between males and females of each species (*F*_2,165_ = 0.18, *p* = .671), suggesting that host sex does not modulate the effects of nematodes on host growth and body size.

**TABLE 1:** Statistical results for the analyses of body size, eclosure time, and survival.

(A) Thorax width						
	Df	SS	MS	*F*	*p*	$\eta_{p}^{2}$
Block	1	8.42	8.42	216.67	<.001	0.07
Species	2	81.05	40.52	1042.84	<.001	0.92
Sex	1	0.90	0.90	23.20	<.001	0.12
Treatment	1	0.22	0.22	5.57	.019	0.02
Species × Sex	2	1.38	0.69	17.76	<.001	0.17
Residuals	168	6.53	0.04			

(B) Eclosure time						
	Df	SS	MS	*F*	*p*	$\eta_{p}^{2}$
Species	2	2477.82	1238.91	469.75	<.001	0.93
Sex	1	10.90	10.90	4.13	.046	0.05
Treatment	1	1.37	1.37	0.52	.473	0.01
Residuals	73	192.53	2.64			

(C) Survival						
	X^2^	Df	*p*			
Block	15.15	1	<.001			
Species	32.25	2	<.001			
Treatment	0.28	1	.598			

*Note*: Tables A and B show ANOVA tables for the effect testing species, sex, and treatment on thorax width and eclosure time, respectively. Table C shows the results of a generalized linear model with binomial error distribution. Non‐significant interaction terms were removed. Partial eta square is shown as an estimate of effect size.

**FIGURE 2:**
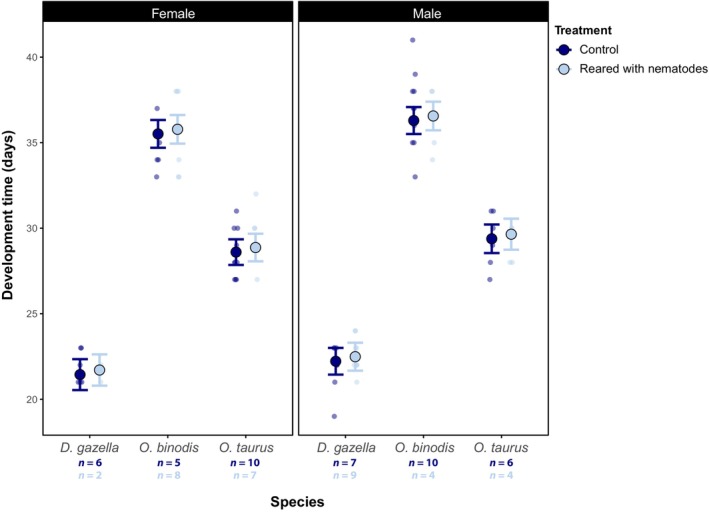
Estimated marginal means of development time across species and treatments for females and males. While egg‐to‐adult development time varies strongly between species, the presence of *Diplogastrellus* nematodes in the larval environment has no effect on the duration of juvenile development. Error bars indicate standard errors associated with parameter estimates derived from the model with the lowest AICc. Sample sizes (*n*) are given by treatment combination.

**FIGURE 3:**
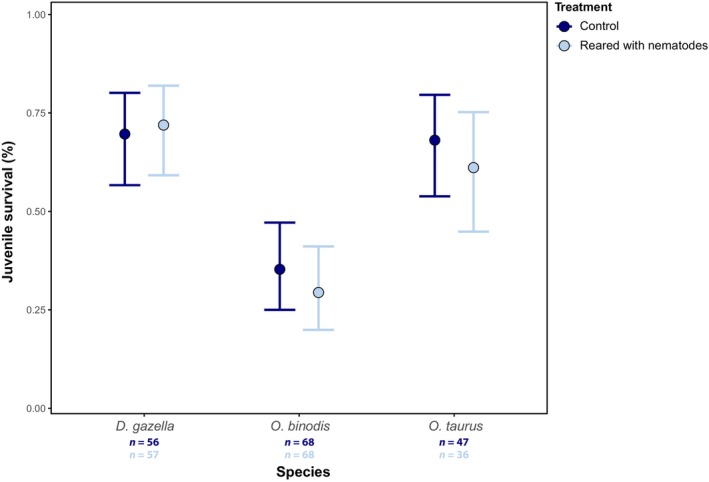
Proportion of individuals that survived to adulthood in each treatment group. The means for each group are demonstrated along with the corresponding 95% binomial confidence intervals. Sample sizes (*n*) are given by treatment combination.

In contrast to body size, we found no significant difference in eclosure time between treatment groups (*F*_1,73_ = 1.37, *p* = .473; Figure 2, Table 1b). This indicates that the presence of nematodes increases dung beetle growth rate as individuals with nematodes grew larger over the same time period. Lastly, species differed strongly in survival (χ^2^_(2)_ = 32.25, *p* < .001), a pattern that was mostly driven by low survival in *O. binodis* (Figure 3). However, we found that the presence of *Diplogastrellus* nematodes did not significantly alter species‐specific survival to adulthood (χ^2^_(1)_ = 0.28, *p* = .598; Figure 3). Taken together, our results show that the presence of *D. monhysteroides* nematodes affects body size but not other fitness‐related traits in a non‐sex‐specific manner across host species.

## 4 DISCUSSION

Sexually transmitted symbionts can have beneficial effects on their hosts (e.g., the GB virus in humans [Bhattarai & Stapleton, 2012]). Yet how widespread such interactions are, and how they may shape host development and evolution remains poorly understood (Smith & Mueller, 2015). Here, we replicate previous findings demonstrating modest yet significant beneficial effects of symbiotic nematodes on body size found in *O. taurus* and show that similar effects are found in other species. However, we did not find any differences in the extent to which nematodes benefit host performance across different host species or host sexes (non‐significant species‐by‐treatment and sex‐by‐treatment interactions), suggesting that the role that mutualistic nematodes play in their host's growth and development may be conserved. Furthermore, we found no indication that the presence of nematodes affected other life history components such as juvenile survival or age at adult eclosure. We discuss the potential prevalence and significance of beneficial sexually transmitted symbionts in insect development and evolution.

### 4.1 Effect of mutualist nematodes on development, fitness, and evolution

Symbionts emerge as important determinants of host development, performance, and fitness (Rosshart et al., 2017). In cases where mutualists are transmitted sexually, this should have major effects on evolutionary and behavioral ecology. For instance, if mating leads to the acquisition of beneficial (or detrimental) symbionts, this should modulate the net benefits of multiple mating and thus shape sexual selection and the evolution of promiscuity (Lombardo et al., 1999; Smith & Mueller, 2015). Our findings show that the presence of *D. monhysteroides* nematodes, which is associated with a large number of functionally important dung beetle species (Sudhaus & Fürst von Lieven, 2003), has positive effects on the size of three dung beetle species. Because these host species diverged around 35–40 million years ago, and the clade derived from their common ancestor now includes a very large number of species (the genus *Onthophagus* alone contains 2000–2500 species [Philips, 2016]), *Diplogastrellus* nematodes may play a major role in dung beetle ecology. However, the extent to which *Diplogastrellus* is found in association with dung beetles, and under which conditions, remains to be investigated. Future research should also assess whether nematodes and dung beetles may be undergoing parallel diversification (phylosymbiosis), as has previously been documented for dung beetle *microbial* symbionts (Parker et al., 2020) or bark beetles and their symbiotic nematodes (Susoy & Herrmann, 2014).

### 4.2 Lack of sex‐specific responses to the presence of symbiotic nematodes

In insects, males and females of the same species commonly differ in their plastic response to nutritional quality and quantity (Stillwell et al., 2010). These sex differences can arise through sex‐specific requirements of limited nutrients but may also be driven by sexual selection favoring stronger (or weaker) responses in a particular sex (Rohner & Blanckenhorn, 2018). The presence of *D. monhysteroides* predictably biases the bacterial and fungal communities contained within brood balls in favor of microbial taxa shown in other systems to aid in the breakdown of otherwise hard‐to‐digest macromolecules such as cellulose (Ledón‐Rettig et al., 2018). *D. monhysteroides* is thus thought to exert its positive effects on dung beetle development through its effects on the microbial environment within which developing larvae are imbedded, which may indirectly affect the availability or accessibility of nutrients (Ledón‐Rettig et al., 2018). However, the exact mechanisms by which this bias in microbial community composition and associated effects on nutrient availability to the host are made possible remain to be identified. Nevertheless, we expected host sexes to differ in their responses to the presence/absence of nematodes yet were unable to recover any support for this hypothesis. This was unexpected because one of the species, *D. gazella*, has previously been shown to exhibit sex‐specific plasticity in size in response to nutrient availability (Rohner, 2021). This raises the possibility that nutritional effects on development may themselves be diverse, only some of which may be influenced by the presence of symbiotic nematodes. Investigating the mechanisms by which some nutritional conditions yield sex‐specific effects while other do not represents an exciting opportunity for future research.

### 4.3 Net costs or benefits of genital worms

If the presence of nematodes increases fitness, nematodes are sexually transmitted, and passed on from parents to offspring, nematodes should spread rapidly within a host population. In addition, selection should favor the evolution of mechanisms to increase the fidelity of transmission, for instance through the evolution of increased mating rate. However, Ledón‐Rettig et al. (2018) report that the proportion of *O. taurus* hosts carrying *D. monhysteroides* nematodes in wild populations was only 0.12 for females and 0.33 for males (with a total sample size of 41 beetles). Although we currently lack comprehensive data for other species and populations, this suggests that the mutualism may be more complex and potentially unstable or context‐dependent. One possibility would be that the phoretic dauer larvae are lost due to environmental stress (also see below). Alternatively, net selection favoring the spread of the symbiosis may be relatively weak. For instance, the benefits of acquiring nematodes through increased mating rates might be counteracted by the risk of co‐infection with other sexually transmitted symbionts (which is common for STIs in various species [Dicker et al., 2003; Lenzko et al., 2011]). This may include microbes, other nematode species, or mites that have negative fitness consequences outweighing the potential benefits of *Diplogastrellus* acquisition. Whether or not co‐infection dynamics may explain the low proportion of adults carrying *Diplogastrellus* nematodes will require further research.

### 4.4 A novel route through which veterinary pharmaceuticals may impair dung beetle ecosystem services

Dung beetles provide vital ecosystem services through, for example, the removal of dung, aeration of the soil, and outcompeting biting and pathogen‐carrying fly species (Losey & Vaughan, 2006). All these effects are dependent on beetle body size (e.g., Nervo et al., 2014; Simmons & Emlen, 2008). Through its effects on host size, the presence of *Diplogastrellus* symbionts may thus indirectly enhance ecosystem services. However, this function is likely to be disrupted in managed agricultural grasslands. Many species of nematodes are common parasites of animals and often cause disease and loss of productivity. Anthelmintic drugs, such as ivermectin, are thus widely used in the treatment of livestock, pets, and humans. While effectively deworming the treated individual, remnants of these drugs (and other antibiotics) can persist in and be excreted in the feces, which exposes the dung insect community to pharmaceutical residues. Such ivermectin residues can have drastic lethal and sublethal effects on various species of dung beetles and dung flies (Conforti et al., 2018; Pérez‐Cogollo et al., 2015; Rodríguez‐Vivas et al., 2020; van Koppenhagen et al., 2020). The presence of mutualist nematodes suggests that some of the sublethal effects of ivermectin exposure on dung beetle growth and adult size may be indirectly caused by the loss of symbiotic nematodes. Future research on deworming drugs' effects on dung beetles and their nematode symbionts is needed to assess the merits of this hypothesis.

### 4.5 Conclusions

Some sexually transmitted symbionts have beneficial effects on their hosts, but the evolutionary significance of these mutualisms remains poorly understood. We show that *Diplogastrellus* nematodes confer modest benefits on the growth of three dung beetle species separated by considerable phylogenetic distances. Our results thus raise the possibility that *Diplogastrellus* nematodes may commonly engage in mutualistic interactions with various dung beetle species. If so, mutualist symbionts may constitute a widespread and potentially considerable source of life history variation in natural populations. However, the patterns found here are overall modest and likely context‐dependent. Future research will thus be necessary to uncover the degree and context in which this symbiosis is beneficial or merely commensal.

## AUTHOR CONTRIBUTIONS

**Levi W. Burdine:** Conceptualization (equal); data curation (equal); formal analysis (equal); methodology (supporting); software (equal); visualization (lead); writing – original draft (supporting); writing – review and editing (supporting). **Armin P. Moczek:** Conceptualization (equal); funding acquisition (equal); resources (lead); writing – original draft (supporting); writing – review and editing (supporting). **Patrick T. Rohner:** Conceptualization (lead); data curation (supporting); formal analysis (equal); funding acquisition (supporting); methodology (equal); project administration (equal); supervision (equal); writing – original draft (lead).

## CONFLICT OF INTEREST STATEMENT

The authors declare no conflict of interest.

## Supporting information


Figure S1.
Table S1.

## Data Availability

Data and R code underlying this study are available on Dryad: https://doi.org/10.5061/dryad.j3tx95xnn.
