# Supplementary material for: Sexually transmitted mutualist nematodes shape host growth across dung beetle species
Source: Ecol Evol. 2024 Mar 10;14(3):e11089. doi: 10.1002/ece3.11089 (PMC10925520; doi:10.1002/ece3.11089)
Supplement: Supplementary file 1 — Figure S1. Table S1. [file ECE3-14-e11089-s001.docx]

**Supplementary Material**

**Sexually transmitted mutualist nematodes shape host growth across dung beetle species**

Levi W. Burdine^1^, Armin P. Moczek^1^, Patrick T. Rohner^1,2*^

^1^Department of Biology, Indiana University Bloomington, Bloomington, IN 47405, United States.

^2^Department of Ecology, Behavior, and Evolution, University of California San Diego, La Jolla, CA 92093, United States

*Corresponding author: Patrick T. Rohner, Department of Ecology, Behavior, and Evolution, University of California San Diego, La Jolla, CA 92093, United States. [prohner@ucsd.edu](mailto:prohner@ucsd.edu).

ORCIDs: LWB: 0009-0001-4724-3574, APM: 0000-0002-3478-9949, PTR: 0000-0002-9840-1050

**
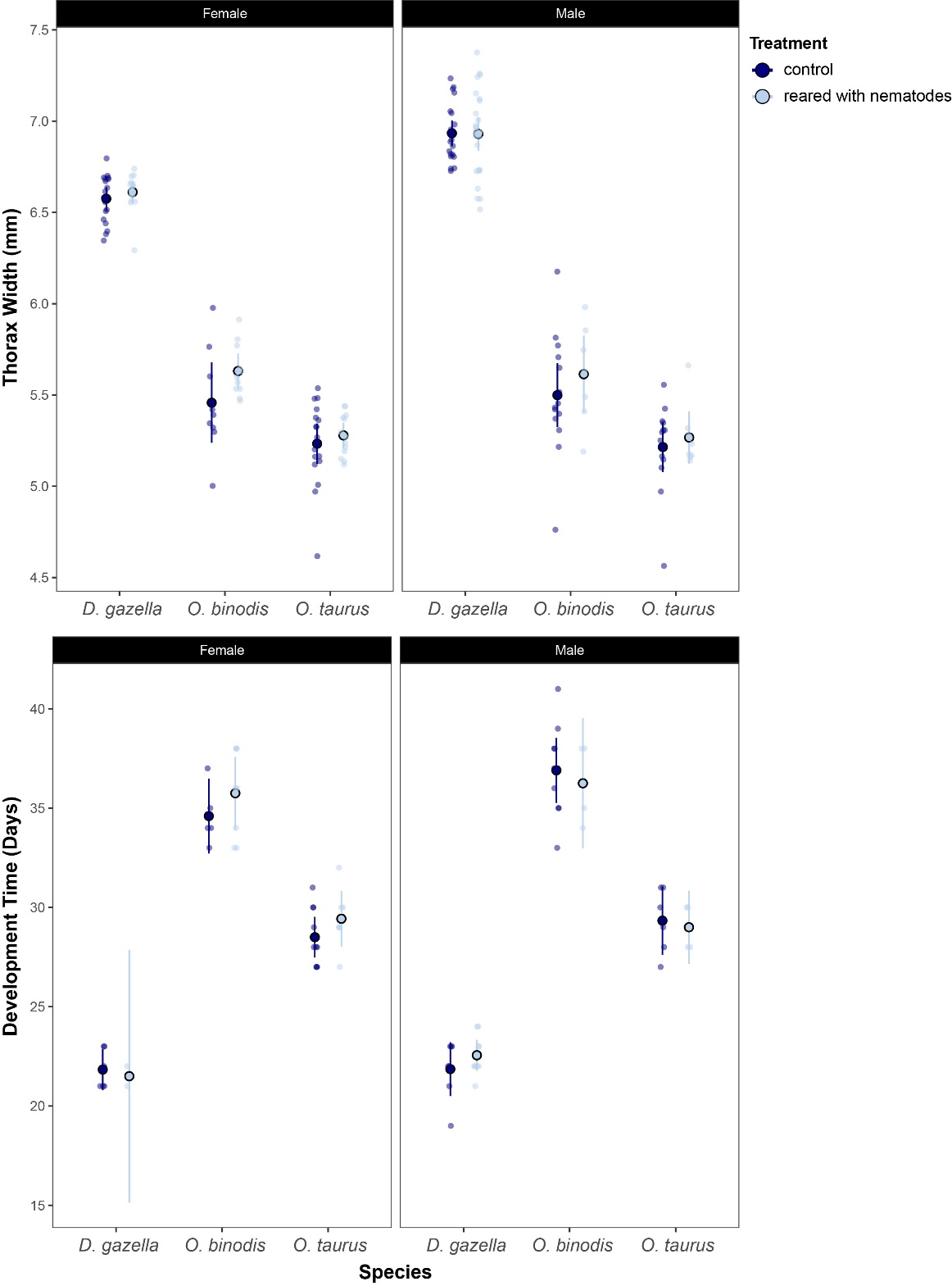
**

**Figure S1.** Raw data on body size (top) and development time (bottom). Bars represent 95% confidence limits estimated based on the observations within group.

Table S1: Model selection table ranking alternative models ordered by AICc. All models included an intercept (Int), experimental block (Blc), sex (Sex), and species (Spc). Models differed in the presence of a treatment (Trt) effect and the presence of interactions.

| model | df | logLik | AICc | delta AICc |
| --- | --- | --- | --- | --- |
| Int+Blc+Sex+Spc+Trt+Sex:Spc | 9 | 40.11 | -61.3 | 0.00 |
| Int+Blc+Sex+Spc+Sex:Spc | 8 | 38.69 | -60.5 | 0.74 |
| Int+Blc+Sex+Spc+Trt+Sex:Spc+Spc:Trt | 11 | 41.94 | -60.3 | 0.99 |
| Int+Blc+Sex+Spc+Trt+Sex:Spc+Sex:Trt | 10 | 40.32 | -59.3 | 1.96 |
| Int+Blc+Sex+Spc+Trt+Sex:Spc+Sex:Trt+Spc:Trt | 12 | 42.03 | -58.2 | 3.10 |
| Int+Blc+Sex+Spc+Trt+Sex:Spc+Sex:Trt+Spc:Trt+Sex:Spc:Trt | 14 | 42.29 | -54.0 | 7.28 |
| Int+Blc+Sex+Spc+Trt | 7 | 23.29 | -31.9 | 29.34 |
| Int+Blc+Sex+Spc+Trt+Spc:Trt | 9 | 25.00 | -30.9 | 30.34 |
| Int+Blc+Sex+Spc+Trt+Sex:Trt | 8 | 23.29 | -29.7 | 31.53 |
| Int+Blc+Sex+Spc | 6 | 20.92 | -29.3 | 31.92 |
| Int+Blc+Sex+Spc+Trt+Sex:Trt+Spc:Trt | 10 | 25.00 | -28.7 | 32.58 |
